# Prevalence of anthelmintic resistance on Lithuanian sheep farms assessed by in vitro methods

**DOI:** 10.1186/s13028-015-0179-y

**Published:** 2015-12-16

**Authors:** Tomas Kupčinskas, Inga Stadalienė, Mindaugas Šarkūnas, Vita Riškevičienė, Marian Várady, Johan Höglund, Saulius Petkevičius

**Affiliations:** Department of Infectious Diseases, Veterinary Academy, Lithuanian University of Health Sciences, Tilžės 18, LT-47181 Kaunas, Lithuania; Institute of Parasitology, Slovak Academy of Sciences, Hlinkova 3, Košice, 04001 Slovak Republic; Department of Biomedical Sciences and Veterinary Public Health, Swedish University of Agricultural Sciences, P.O. Box 7028, SE-750 07 Uppsala, Sweden

**Keywords:** Anthelmintic resistance, In vitro, Sheep nematodes, Egg hatch test, Larval development test

## Abstract

**Background:**

This study examines the prevalence of drug resistance in gastrointestinal nematodes to macrocyclic lactones (ML) and benzimidazoles (BZ) in Lithuanian sheep using sensitive and precise in vitro methods. The survey was conducted from August 2013 to November 2014. Thirty-three farms with sheep previously treated with BZ and ivermectin (IVM) were included in the study. On 12 farms where only BZ were used, egg hatch discrimination dose testing (EHDDT) was conducted to detect anthelmintic resistance (AR) to BZ. On eight farms where only ML were used, micro agar larval development testing (MALDT) was conducted to detect AR to ivermectin (IVM). On the remaining 13 farms, where both classes of drugs were used, EHDDT and MALDT were both applied to detect multidrug resistance to BZ and IVM.

**Results:**

BZ-resistant gastrointestinal nematodes were found on all 25 farms with a previous history of BZ use. High levels of resistance (>40 % of hatching) were recorded on 36 % of these farms, and low levels (<20 % of hatching) on 40 % of farms. IVM-resistant populations were found on 13 out of 21 sheep farms using this drug. Of these 13 farms with AR to IVM, low levels of resistance (<30 % development) were recorded on 84.6 % of farms and high levels (>30 % development) on 15.4 % of farms. No resistance to IVM was recorded on 38.1 % of farms. Multi-drug resistance was detected on five farms out of 13 (38.5 %) using both classes of drugs.

**Conclusions:**

The present study demonstrates the existence of AR to BZ and ML on Lithuanian sheep farms thus confirming results in a previous in vivo study. Cases of multi-drug resistance were recorded in the present study and require further consideration. An appropriate strategy for anthelmintic treatment, measures to prevent gastrointestinal nematode infection and a better understanding of the management practices associated with resistance may slow down further development of AR.

## Background

Gastrointestinal nematode (GIN) infections remain the most prevalent parasitic diseases and are controlled on many farms with continuous anthelmintic treatments [1]. Sheep farmers mainly use benzimidazoles (BZ) and macrocyclic lactones (ML) for chemotherapy, but overuse of anthelmintic drugs leads to the selection of resistant parasites [2]. The rate of emergence of resistant strains has generally been lower in temperate zones in the northern hemisphere compared with other regions due to different climate, parasite species and treatment frequency [3]. However, occurrence of resistance in Europe is increasing and anthelmintic resistance (AR) not only to a single drug, but resistance to two or even all three major anthelmintic groups (multidrug resistance) has also been reported [4–6]. AR to both BZ and ML has been recorded in the Slovak Republic [7], Spain [8, 9], Italy [10, 11], Greece [12], the United Kingdom [13, 14] and the Netherlands [15].

AR to BZ is widespread in Europe, with high levels of resistance, while AR to ML is relatively low [16]. Low levels of ivermectin (IVM) resistance may be misdetected with the current most widely used method, the faecal egg count reduction test (FECRT) [17]. The accuracy of this method depends on a correlation between egg counts and worm burdens, which is not always the case [18]. Therefore determination of AR has to be performed using more efficient methods, e.g. in vitro tests [1, 19].

In a recent study using FECRT [20], AR was detected in 27.8 % of Lithuanian sheep flocks irrespective of the anthelmintic used. In that study, 71.8 % of sheep farmers surveyed used anthelmintics against GINs and the most commonly used classes of anthelmintics were ML (68.6 %; 95 % CI 56.9–8.2), BZ (27.5 %; 95 % CI 18.1–38.6) and levamisole (3.9 %; 95 % CI 0.9–9.8) [20]. However, information about the prevalence of GIN in Lithuanian sheep flocks is limited and the AR reported in vivo has not been confirmed by in vitro methods. The aim of the present study was therefore to examine the prevalence of drug resistance in GIN species to ML and BZ in Lithuanian sheep flocks using cheap, sensitive and precise in vitro methods, which also have the potential to detect low levels of resistance.

## Methods

### Trial design

The survey was conducted from August 2013 to November 2014. A total of 33 farms, mainly in central and southern Lithuania, were enrolled in the study. All tfarms had a history of use of fenbendazole and/or IVM for 3–5 years and the last anthelmintic treatment was carried out at least 10 weeks before the start of the study. On 12 of the 33 farms, where only BZ were used, an egg hatch discrimination dose test (EHDDT) was conducted for detection of AR to BZ. On eight other farms where only ML were used, a micro agar larval development test (MALDT) was conducted for detection of AR to IVM. On the remaining 13 farms, where both classes of drugs were used, both tests were applied to test for multidrug resistance to BZ and IVM.

The size of sheep flocks was 40–1000 animals per farm and most farms had Lithuanian black-headed sheep and crossbreed sheep. On all farms, the animals grazed on pasture from April to October. On each farm, faeces samples were taken from the rectum of 15–20 randomly selected animals. Pooled faeces samples weighing 50–100 g were stored anaerobically in plastic tubes filled with water at room temperature and processed during the next 2 days [21]. Nematode eggs were isolated by sequential sieving of the faeces through three stacked sieves with mesh size 20, 100 and 250 µm. The material collected on the 20 µm sieve was washed with water and sedimented by centrifugation. Trichostrongylid eggs were then recovered by the sugar flotation method and used for in vitro EHDDT and/or MALDT [22, 23].

The study was performed in compliance with Lithuanian animal welfare regulations (No. B1-866, 2012; No. XI-2271, 2012) and was approved by the Lithuanian Committee of Veterinary Medicine and Zootechnic Sciences (Protocol No.07/2010).

### Egg hatch discrimination dose test

To examine AR to BZ, EHDDT was performed as described by Coles et al. [24]. A stock solution of thiabendazole (TBZ) (Sigma-Aldrich, Germany) was prepared by dissolving the pure compound in dimethyl sulphoxide (DMSO) (Sigma-Aldrich, Germany). The final concentration was prepared by adding 10 µl of the TBZ solution to 1.99 ml of an aqueous suspension with approximately 150 eggs/ml [21]. For the purposes of this study, EHDDT was only used at the single working concentration of 0.1 µg/ml. A control, 0.5 % DMSO solution without anthelmintic, was also included in the test. The egg suspensions were dispensed into 24-well plates (Nuncleon, Denmark) and incubated at 27 °C for 48 h. The test was stopped by adding 10 µl of Lugol’s iodine and the first 100 eggs and/or larvae were counted in each well. The test was performed with two replicates. The results on AR to BZ i were interpreted according to the method described by Dolinska et al. [21].

### Micro-agar larval development test

The MALDT was performed in 96-well microtitre plates as described by Coles et al. [25]. Ivermectin aglycone (IVM-Ag) was chosen for this test because of the higher ability to differentiate between the IVM-resistant and susceptible isolates [26]. Stock solutions of IVM-Ag were serially diluted 1:2 with DMSO to produce 12 concentrations ranging from 0.084 to 173.6 ng/ml. Then 12 μl of each stock solution with different final concentrations were mixed with 150 μl of 2 % Bacto agar (Difco, USA) and stored at 4 °C for 5 min. To inhibit fungal growth, 10 μl of eggs (final number of 50 eggs per well) in a 0.3 mg/ml solution of amphotericin B (Sigma-Aldrich, Germany) were mixed with 10 μl of yeast extract and added to the agar [21, 23]. Yeast extract was prepared as described by Hubert and Kerboeuf [27]. Only DMSO (1.3 %) was used in the control wells. The plates were incubated for 7 days at 27 °C. Incubation was terminated by adding Lugol’s iodine solution to each well. After incubation, the proportions of unhatched eggs, L_1_–L_2_ and L_3_ stage larvae at each concentration were determined under an inverted stereomicroscope. For IVM resistance, a threshold discriminating concentration of 21.6 ng/ml was chosen and the results were interpreted according to the method described by Dolinska et al. [26, 28].

### Statistical analysis

The lower and higher limits of the 95 % confidence interval were calculated following WAAVP recommendations [24]. Descriptive statistics were calculated using Microsoft^®^ Excel 2007 and IBM SPSS Statistics (Version 21.0). Differences in the level of resistance to BZs and MLs were calculated using Fisher’s exact test, performed using GraphPad Prism version 4.00.

## Results

**Fig. 1:**
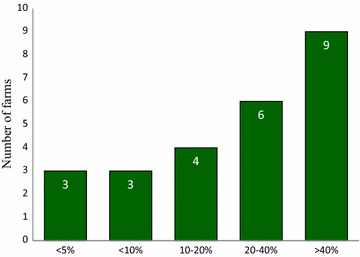
Number of sheep farms with different percentage levels of hatched eggs at a threshold of 0.1 μg/ml thiabendazole detected by EHDDT on 25 farms

Instead of using the conventional threshold values (ED_50_ or ED_99_), the number of hatched eggs at a discrimination dose (DD) concentration of 0.1 µg/ml was used, because DD prevents 99 % of the susceptible eggs from hatching [21]. The percentage of hatched eggs was categorised into low, medium or high based on farm status (susceptible/resistance) determined by hatching in the EHDDT. BZ-resistant GIN were found in all 25 farms investigated. On 36 % of these farms (9/25; 95 % CI 18.0–57.5), a high level of resistance (>40 % of hatching) was recorded. A medium level of resistance in GIN was recorded on 24 % of farms (6/25; 95 % CI 9.4–45.1), while a low level of resistance (<20 % of hatching) was recorded on 40 % of farms (10/25; 95 % CI 21.1–61.3) (Fig. 1). However the differences in incidence of different levels of resistance were not significant (P > 0.05).

**Fig. 2:**
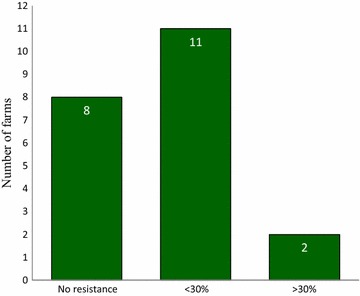
Number of sheep farms with development of larvae at a threshold of 21.6 ng/ml ivermectin aglycone detected by MALDT on 21 farms

The results for IVM resistance among GIN determined using MALDT in vitro on 21 sheep farms are presented in Fig. 2. On 61.9 % of these farms (13/21; 95 % CI 38.4–81.9), L_3_ larvae had developed at the threshold concentration of 21.6 ng/ml. No resistance to IVM was detected on 38.1 % of farms (8/21; 95 % CI 18.1–61.6), all of which were in the group of farms where both classes of anthelmintics were used. The percentage of developed L_3_ larvae at the threshold concentration was categorised as low (<30 % development of larvae) or high (>30 %). The differences between groups where AR to IVM was detected and no resistance to IVM was found were not significant (P > 0.05). A low level of resistance was detected on 84.6 % of farms (11/13; 95 % CI 54.6–98.1) (P < 0.05), while a high level of resistance was recorded on 15.4 % of farms (2/13; 95 % CI 1.9–45.4). The differentiation of L_3_ larvae at the discrimination concentration in the MALDT revealed the presence of *Teladorsagia/Trichostrongylus* spp. in all tests.

Low levels of multidrug resistance were detected on five (38.5 %; 95 % CI 13.9–68.4) out of 13 sheep farms. From this group, where both classes of anthelmintics were used, AR to BZ was found on all farms, while AR to IVM-Ag was found on only five out of 13 farms.

## Discussion

A previous study on AR on Lithuanian sheep farms using in vivo FECRT reported 20 % (95 % CI 4.3–48.1) prevalence of AR to BZ on three out of 15 farms, while AR was suspected on one farm (6.7 %; 95 % CI 0.2–31.9) [20]. However, the correlation between FECRT and EHT, on which detection of BZ resistance is based, is sometimes low [1, 29]. As an alternative, in vitro methods are cheaper, faster, more sensitive and suitable for widespread use in field screening surveys.

The results from the present in vitro study show that AR to BZ and ML is prevalent on the sheep farms studied, irrespective of the anthelmintic used. However, the results of this in vitro study and those of the previous in vivo study [20] cannot be statistically compared, due to different farms being enrolled in the two studies. Using in vitro EHDDT, AR to BZ was found on all farms studied, but there was no significant difference (P > 0.05) between the occurrence of high (36 %), medium (24 %) and low (40 %) levels of AR.

Despite the high prevalence of AR to BZ on the Lithuanian sheep farms studied, the levels of resistance were still low and/or medium (<40 % of hatching). A possible explanation for the high prevalence of AR to BZ observed on these sheep farms is the long history of BZ availability on the Lithuanian market. Lack of rotation of anthelmintics could also be responsible for the increasing development of BZ resistance, as anthelmintics were found to be rotated on only 4.8 % of sheep farms in the previous study [20].

In general, BZ resistance is much more prevalent in some, but not all, countries compared with IVM resistance [30]. Studies using different in vivo and in vitro methods have recorded AR to BZ on 83 % of sheep farms examined in western France [18], 11.0 % in Norway [31], 13.6 % in Spain [6] and 100 % in Slovak Republic [21].

The previous study in Lithuania using in vivo FECRT found AR to IVM on 12.5 % of farms studied [20]. A comparable prevalence of AR to IVM, which is much lower than the prevalence of AR to BZ, has been reported for other European countries [11, 21]. Based on the results of the present study, AR to IVM has not yet reached a critical level as a low level of AR to IVM was recorded on the majority of sheep farms (84.6 %; P < 0.05), while a high level of AR was only recorded on the remaining 15.4 % of farms. No AR to IVM was recorded on over one-third of sheep farms (38.1 %). Ivermectin is the most used anthelmintic on Lithuanian sheep farms (68.6 %) [20]. The lower price of IVM and the easier and faster administration compared with fenbendazole, which is administered orally, are the main reasons for the selection of injectable ivermectin on sheep farms [20]. Although IVM is quite a new drug used on Lithuanian sheep farms, frequent use of IVM in combination with clorsulon for the treatment of GIN and *Fasciola hepatica* in sheep could make some contribution to the development of AR to IVM. In addition to underdosing and lack of rotation of drugs, lack of strict quarantine for newly introduced breeding animals and low knowledge of their treatment history with anthelmintics have been recorded. Moreover, knowledge about AR, the prevalence of GINs and proper usage of anthelmintics is also limited among Lithuanian sheep farmers [20].

Data on AR obtained using in vitro methods can be analysed using the conventional threshold values ED_50_/LC_50_ or ED_99_/LC_99_ [32]. However, EHDDT and MALDT using the ED_99_/LC_99_ criterion and the threshold DD have the potential to detect low levels of resistance [33], although the ED_50_/LC_50_ criterion is not able to provide early detection during the development of resistance [21]. In addition, EHDDT provides a good estimate of genotype resistance [34]. Analysing data using a threshold discriminating concentration of 0.1 µg/ml for thiabendazole and 21.6 ng/ml for IVM-Ag is faster, simpler and inexpensive [26, 28]. In addition, if ED_50_/LC_50_ or ED_99_/LC_99_ could be used to describe the results, the number of farms with AR would probably decrease, but using the DD criterion is more sensitive and can specifically reveal a small proportion of resistance in the population.

The multidrug resistance detected in the present study is of increasing concern in Lithuania and in many other countries. Multidrug resistance has been reported in Brazil [35], New Zealand [36], the United Kingdom [37], Germany [5] and Spain [6]. The rapid evolution of multiple anthelmintic resistance may lead to total anthelmintic failure [1, 38, 39]. The first reported case of resistance to monepantel, a novel drug class which first became available in New Zealand in 2009, is another example of the fast development of AR [40, 41].

In the present study, low and/or medium levels of multidrug resistance were detected with both tests (e.g. 16.2 % development of larvae to L_3_ at a threshold of 21.6 ng/ml IVM-Ag in MALDT and 14.0 % of hatched eggs at a threshold of 0.1 µg/ml TBZ in EHDDT). The in vitro tests greatly improved knowledge on the level of AR in Lithuanian sheep farms, showing markedly higher occurrence of AR than previous reports based on in vivo methods [20, 42].

## Conclusions

This study demonstrated the existence of resistance to BZ and ML on Lithuanian sheep farms, confirming previous results from in vivo studies. Cases of multi-drug resistance were also demonstrated and require further consideration. An appropriate strategy for anthelmintic treatment, measures to prevent GIN infections and a better understanding of management practices associated with resistance could slow the development of resistance.
